# Genome-wide identification, expression analysis and functional study of the *GRAS* gene family in Tartary buckwheat (*Fagopyrum tataricum*)

**DOI:** 10.1186/s12870-019-1951-3

**Published:** 2019-08-06

**Authors:** Moyang Liu, Li Huang, Zhaotang Ma, Wenjun Sun, Qi Wu, Zizhong Tang, Tongliang Bu, Chenglei Li, Hui Chen

**Affiliations:** 1 College of Life Science, Sichuan Agricultural University, Ya’an, China; 2 School of Agriculture and Biology, Shanghai Jiao Tong University, Shanghai, China

**Keywords:** Tartary buckwheat, *GRAS*, Genome-wide, Fruit development, Expression patterns

## Abstract

**Background:**

GRAS are plant-specific transcription factors that play important roles in plant growth and development. Although the *GRAS* gene family has been studied in many plants, there has been little research on the *GRAS* genes of Tartary buckwheat (*Fagopyrum tataricum*), which is an important crop rich in rutin. The recently published whole genome sequence of Tartary buckwheat allows us to study the characteristics and expression patterns of the *GRAS* gene family in Tartary buckwheat at the genome-wide level.

**Results:**

In this study, 47 *GRAS* genes of Tartary buckwheat were identified and divided into 10 subfamilies: LISCL, HAM, DELLA, SCR, PAT1, SCL4/7, LAS, SHR, SCL3, and DLT. *FtGRAS* genes were unevenly distributed on 8 chromosomes, and members of the same subfamily contained similar gene structures and motif compositions. Some *FtGRAS* genes may have been produced by gene duplications; tandem duplication contributed more to the expansion of the *GRAS* gene family in Tartary buckwheat. Real-time PCR showed that the transcription levels of *FtGRAS* were significantly different in different tissues and fruit development stages, implying that *FtGRAS* might have different functions. Furthermore, an increase in fruit weight was induced by exogenous paclobutrazol, and the transcription level of the DELLA subfamily member *FtGRAS22* was significantly upregulated during the whole fruit development stage. Therefore, *FtGRAS22* may be a potential target for molecular breeding or genetic editing.

**Conclusions:**

Collectively, this systematic analysis lays a foundation for further study of the functional characteristics of *GRAS* genes and for the improvement of Tartary buckwheat crops.

**Electronic supplementary material:**

The online version of this article (10.1186/s12870-019-1951-3) contains supplementary material, which is available to authorized users.

## Background

Transcription factors (TFs) bind to specific DNA sequences (cis-acting elements) in the promoters of target genes, which can promote or inhibit the transcription level of the target genes. TFs play an important role in plant growth and development and response to stress (such as drought, heat, cold and salt) [1–3]. GRAS proteins are plant-specific transcriptional regulators that have been found in recent years [4]. GRAS proteins were named after the first three members of the family, gibberellic acid insensitive (GAI), repressor of GA1–3 mutant (RGA), and scarecrow (SCR) [5–7]. The highly conserved region of the C-terminal region of GRAS proteins is commonly referred to as the GRAS domain. Most members of the GRAS family have only one GRAS domain, but a few GRAS proteins have two GRAS domains, or the C-terminus has another functional domain in addition to one GRAS domain [8]. The GRAS domain can be divided into five units: leucine-rich region I (LHRI), VHIID, leucine-rich region II (LHRII), PFYRE, and SAW [9]. Previous studies have found that LHRI and LHRII play a key role in the homologous dimerization of GRAS protein. The VHIID motif is the core structure of the GRAS protein, and P-N-H-D-Q-L in the VHIID motif is very conserved and ends with L-R-I-T-G. The PFYRE motif consists of three parts: P, FY and RE, which may be related to phosphorylation. The SAW motif contains three pairs of conserved amino acid residues R-E, W-G and W-W [9]. However, the N-terminus of GRAS protein is a highly variable region that contains two highly conserved protein structures, DELLA and TVHYNP [10]. The highly variable amino acid sequence of the N-terminus can be folded into a specific molecular recognition structure that binds to the target protein and participates in the signal transduction process [8]. The *GRAS* gene family is usually divided into 10 subfamilies: DELLA, DLT, HAM, PAT1, LAS, LISCL, SCR, SCL3, SHR and SCL4/7 [8]. For the GRAS family that is gradually being identified in other plants, the family classification is slightly different [8–11]. For example, 93 *GRAS* genes in *populus* have been divided into 13 subfamilies (DLT, DELLA, HAM, PAT1, LAS, SHR, LISCL, SCR, SCL3, SCL4/7, Os19, Os4, PT20), of which PT20 are newly discovered [11]. The 50 GRAS genes in pepper are divided into 10 subfamilies (DLT, DELLA, HAM, PAT1, LAS, SCL3, LISCL, SCR, SHR, Os4 and Ca_GRAS), of which Ca_GRAS is a specific subfamily in pepper [2].

As unique transcription factors in plants, GRAS proteins play an important role in plant growth and development [12]. AtSCL13 (PAT1 subfamily) is involved in phytochrome A (phyA) signal transduction, and plays a major role in hypocotyl elongation during the de-etiolation of *Arabidopsis thaliana* [13]. The expression of *GRAS2* (PAT1 subfamily) in tomato was reduced, resulting in a decrease in fruit weight [14]. OsSCR not only participates in the formation of stomata and ligule but also regulates asymmetric division of cells [15]. The *PhHAM* gene in Petunia is mainly expressed in the primordia of lateral organs and stem provascular tissues. The *PhHAM* gene acts on adjacent tissues in a noncellular autonomous way to maintain the activity of the apical meristem [16]. NSP1 (SHR subfamily) and NSP2 (HAM subfamily), form a DNA binding complex to induce gene expression during nodulation signaling in *Medicago truncatula* [17]. The mutation of the *DLT/OsGRAS-32* gene in rice results in the decrease in GA content and plant dwarfing [18]. SCL3 and DELLA balance gibberellin feedback regulation via IDD proteins [19]. In addition to participating in plant growth and development, members of the *GRAS* family are also involved in plant responses to various abiotic stresses. *Gh_A01G0682* and *Gh_A04G0081* are upregulated under salt and PEG stress [20]. *MtGRAS32* and *MtGRAS60* are positively upregulated but *MtGRAS47* and *MtGRAS45* downregulated after GA3 treatment [21]. Until now, the regulatory mechanism of DELLA proteins in the GRAS gene family has been studied extensively and thoroughly. DELLA proteins are the main negative regulators of gibberellin (GA) signal transduction. Research in *Arabidopsis thaliana* suggests that GA regulates late embryo development by regulating DELLA protein levels [22]. By decreasing DELLA activity, it can promote the growth of parthenocarpic fruits in tomato [23]. The interaction between DELLA and ARF/IAA mediates the crosstalk between gibberellin and auxin signaling to control the initiation of tomato fruit [24]. There is only one DELLA protein SLR1 in rice, and GA signals promote cellulose synthesis by relieving the interaction between SLR1 and NACs [25].

Tartary buckwheat is a dicotyledonous plant in the *Polygonaceae* family that contains a variety of nutrients, especially flavonoids such as rutin and quercetin [26]. Until now, transcription factor families such as ARF, MADS, AP2/ ERF, NAC, bZIP, ZF-HD have been identified in Tartary buckwheat [27–32]. More importantly, through in-depth research of *ARF2*, it has been found that *ARF2* plays an important role in determining the final size of Tartary buckwheat fruit [33]. The *GRAS* gene family has been widely studied in many plants, such as *Arabidopsis thaliana*, rice, tomato, pepper, maize and Chinese cabbage [2, 11, 34–37]. However, few studies have examined GRAS proteins in Tartary buckwheat. Due to the important role of *GRAS* genes in various physiological processes, study of the Tartary buckwheat *GRAS* gene family is important. The recent complete genome sequencing of Tartary buckwheat provides researchers an opportunity to reveal the tissue expression profile and evolution of the *GRAS* gene family in Tartary buckwheat [38]. In this study, we first analyzed the gene structure, chromosomal location, duplication events of 47 *FtGRAS* genes, and motif composition, 3D structure of 47 FtGRAS proteins. We then compared the evolutionary relationship with 7 species (*Arabidopsis thaliana*, rice, soybean, grape, tomato, sunflower, beet). Next, the expression patterns of the *FtGRAS* genes in different tissues and different fruit development stages were determined. Finally, we further explored the relationship between DELLA and Tartary buckwheat fruit development. In summary, this research provides valuable clues for the functional characterization of members of the *GRAS* gene family during the growth and development of Tartary buckwheat.

## Results

### Identification of *FtGRAS* genes in Tartary buckwheat

In this study, 47 *FtGRAS* genes were identified from the Tartary buckwheat genome. They were then renamed *FtGRAS1* to *FtGRAS47* according to their chromosomal location (Additional file 3: Table S1). The basic characteristics were analyzed, including the coding sequence length (CDS), protein molecular weight (Mw), isoelectric point (pI) and subcellular localization (http://cello.life.nctu.edu.tw/) (Additional file 3: Table S1). Of the 47 FtGRAS proteins, FtGRAS7 was the smallest protein with 44 amino acids, and the largest protein was FtGRAS1 with 755 amino acids. The Mws of the proteins ranged from 5.19 kDa (FtGRAS1) to 83.5 kDa (FtGRAS7), and the pI ranged from 4.85 (FtGRAS19) to 9.72 (FtGRAS39), with a mean of 6.45. The CDSs of the *FtGRAS* genes varied greatly, ranging from 132 to 2265 bp. The CDS of *FtGRAS7* was the shortest at 132 bp, and the CDS of *FtGRAS1* was the longest, reaching 2265 bp. The predicted subcellular localization results showed that 19 FtGRAS proteins were located in the nuclear region, 14 in the cytoplasm, 8 in the plasma membrane, 3 in the chloroplast, and 3 in the mitochondria.

### Phylogenetic analysis and classification of *FtGRAS* genes

**Fig. 1:**
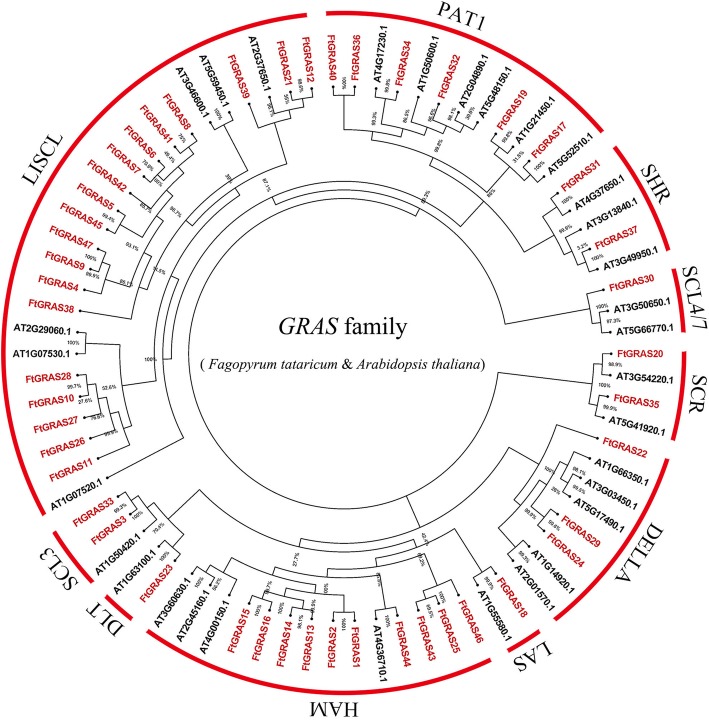
Phylogenetic tree representing the relationships among GRAS of Tartary buckwheat and *Arabidopsis thaliana* used the Maximum Likelihood (ML) method

To explore the phylogenetic relationship of GRAS protein in Tartary buckwheat, we constructed a phylogenetic tree using the Maximum Likelihood (ML) method based on the amino acid sequences of 47 FtGRAS and 31 AtGRAS proteins (Fig. 1). According to their homology with GRAS proteins in *Arabidopsis thaliana*, the 47 *GRAS* genes of Tartary buckwheat were divided into 10 subfamilies: LAS, SCL4/7, HAM, SCR, DLT, SCL3, DELLA, PAT1, SHR, and LISCL. The LISCL subfamily had the largest number of members, with 19 *FtGRAS* genes. The SCL4/7, LAS, and DLT subfamilies all contained only one member, and there were 10, 6, 3, 2, 2, and 2 *FtGRAS* genes in HAM, PAT1, DELLA, SCR, SHR, and SCL3, respectively (Fig. 1).

### Gene structure and motif composition of the *FtGRAS* gene family

**Fig. 2:**
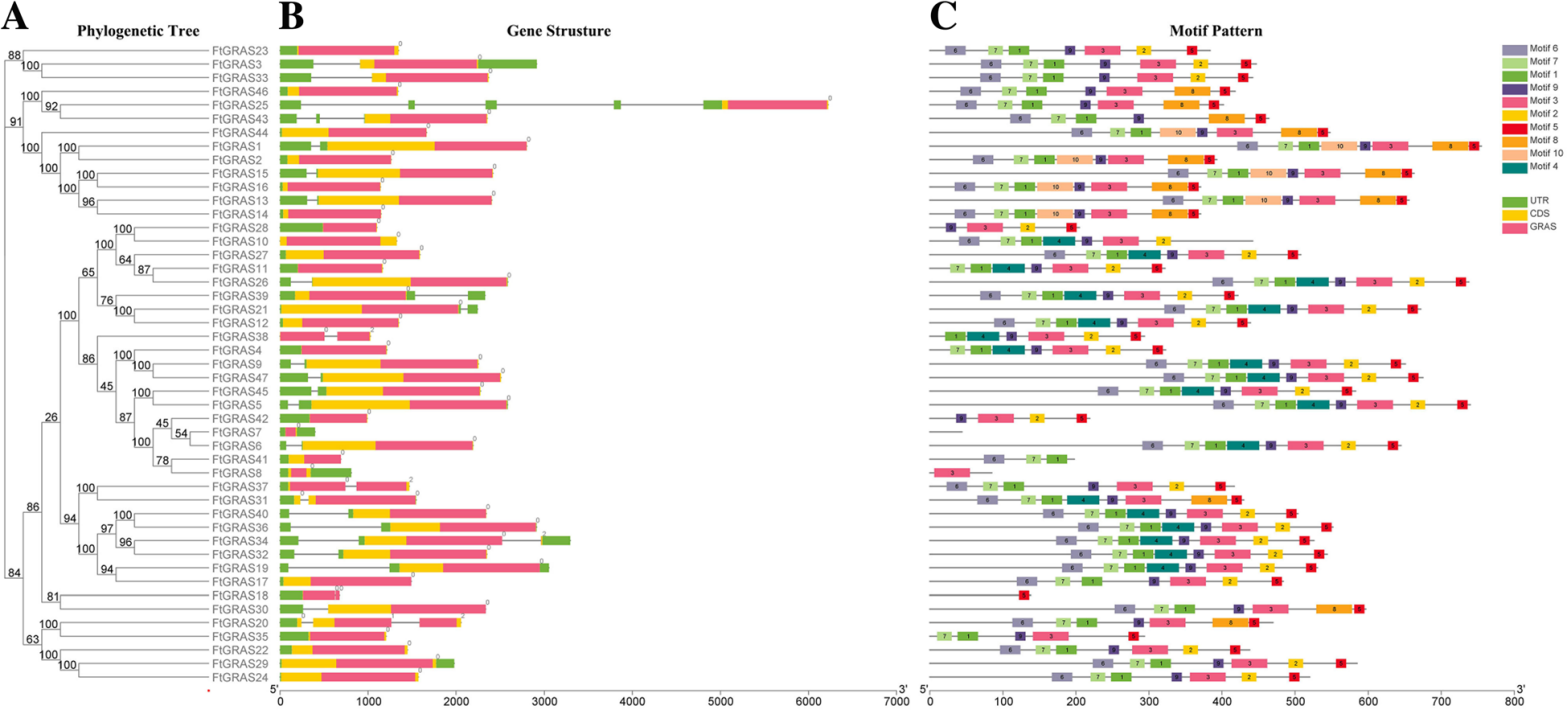
Phylogenetic relationships, gene structures and architecture of the conserved protein motifs in GRAS genes from Tartary buckwheat (**a**) The phylogenetic tree was constructed based on the full-length sequences of Tartary buckwheat GRAS proteins. **b** Exon-intron structure of Tartary buckwheat GRAS genes. Green boxes indicate untranslated 5`- and 3`-regions; yellow boxes indicate CDS, and red boxes indicate GRAS domains. The number indicates the phase of the corresponding introns. **c** Motif composition of the Tartary buckwheat GRAS proteins. The motifs, numbered 1–10, are displayed in different colored boxes. The sequence information for each motif is provided in Additional file 4: Table S2. The length of the protein can be estimated using the scale at the bottom

To understand the structural components of the *FtGRAS* genes, the exon and intron structures of the *FtGRAS* genes were obtained by comparing the corresponding genomic DNA sequences (Fig. 2b). Forty-seven *FtGRAS* genes all contained the GRAS domain, and most of the *FtGRAS* genes (41, ~ 87%) contained no introns; *FtGRAS18, FtGRAS31, FtGRAS34, FtGRAS37* and *FtGRAS38* contained one intron, and only *FtGRAS20* contained two introns. In general, members of the same subfamily had similar gene structures.

To further study the characteristic region of the FtGRAS proteins, the motifs of 47 FtGRAS proteins were analyzed using an online MEME (Fig. 2c). A total of 10 distinct conserved motifs (named Motif 1–10) were found (Fig. 2c; Additional file 4: Table S2). The motifs were arranged according to the sequence of domains, with motif 6 belonging to the LHRI domain, motif 7 and 1 belonging to the VHIID domain, motif 4 and 9 belonging to the LHRII domain, motif 3 belonging to the RFYRE domain, motif 2, 8, 5 belong to the SAW domain. Motif 10 was distributed between motif 1 and motif 4. Most of the FtGRAS proteins (89%) contained motif 3 and motif 5. FtGRAS7 did not contain any motif, FtGRAS8 contained only motif 3, and FtGRAS18 contained only motif 5. Simultaneously, we found that some motifs were only present in specific subfamilies. For instance, motif 4 was only present in LISCL, SHR and PAT1, and motif 10 only in HAM. When the *FtGRAS* gene family members were compared, the results showed that most of the closely related members had similar motifs. For example, the SCL3 group contained motifs 6, 7, 1, 9, 3 2, and 5, but the DELLA group contained motifs 6, 7, 1, 4, 9, 3, and 2.

Protein models of all the 47 FtGRAS were built using SWISS-MODEL (Additional file 1: Figure S1), and the results showed that the tertiary structures of FtGRAS protein mainly contained α-helices and random coils. The six proteins (FtGRAS7, 8, 18, 34, 41, 42) contained fewer α-helices and random coils (Additional file 1: Figure S1). Overall, the conserved motif composition and similar gene structures within the same groups of *GRAS* members, coupled with the results of the phylogenetic analysis, supported the reliability of the population classification.

### Chromosomal distribution and synteny analysis of *FtGRAS* genes

**Fig. 3:**
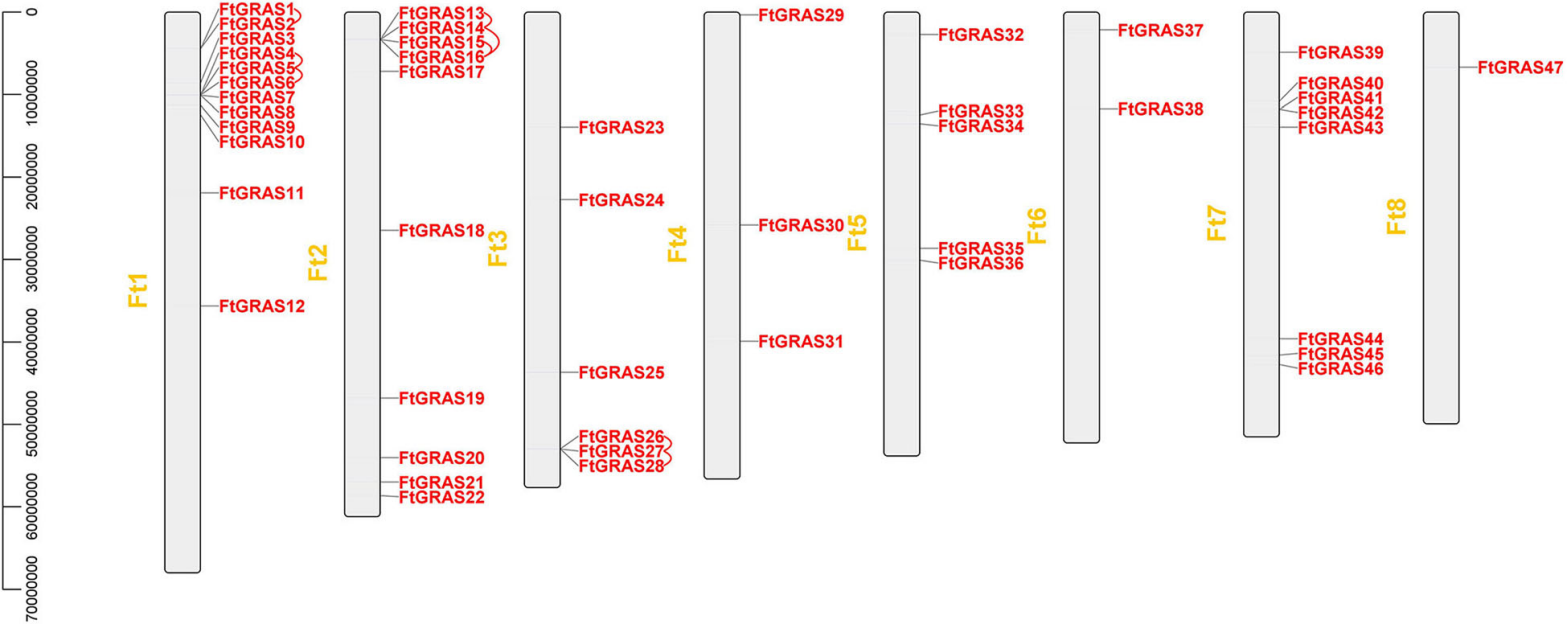
Schematic representations of the chromosomal distribution of Tartary buckwheat *GRAS* genes. The red lines indicate duplicated *GRAS* gene pairs. The chromosome number is indicated to the left of each chromosome

A map of the physical position of the *FtGRAS* genes was created based on the physical location information of the Tartary buckwheat genome (Fig. 3). According to the result, the *FtGRAS* genes were unevenly distributed on 8 chromosomes of Tartary buckwheat. Ft1 had the most *FtGRAS* genes (12, ~ 26%), followed by Ft2 (10, ~ 21%), Ft7 (8, ~ 17%), Ft3 (6, ~ 13%), Ft5 (5, ~ 11%), Ft4 (3, ~ 6%), Ft6 (2, ~ 4%) and Ft8 containing only one *GRAS* gene (~ 2%). Interestingly, the number of *GRAS* genes distributed in the middle of the 8 chromosomes in Tartary buckwheat was relatively low, and the distribution of the *GRAS* gene on the chromosomes was similar to *ATGRAS* and *OsGRAS* [10].

**Fig. 4:**
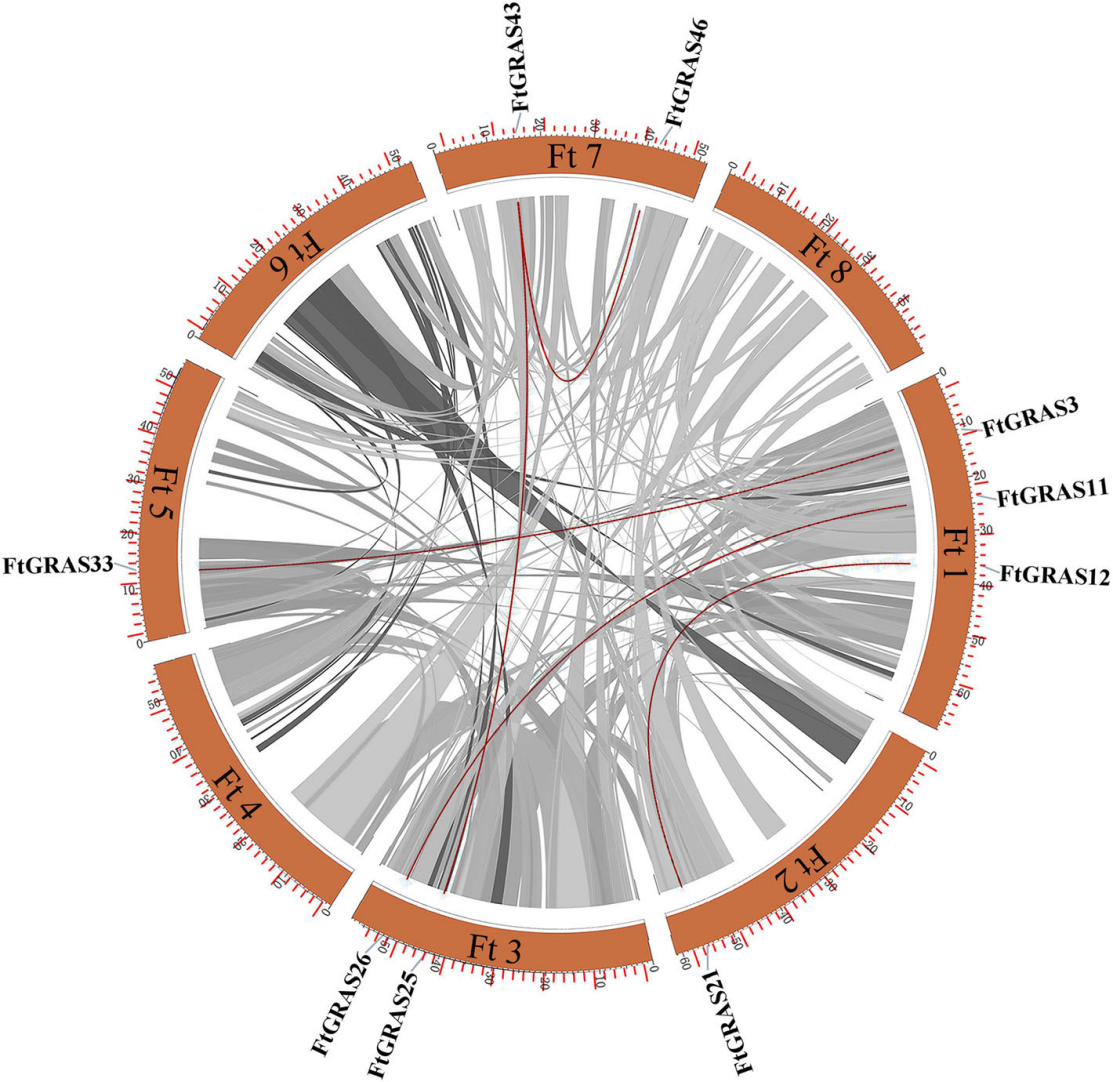
Schematic representations of the inter-chromosomal relationships of Tartary buckwheat *GRAS* genes. Gray lines indicate all syntenic blocks in the Tartary buckwheat genome, and red lines indicate duplicated *GRAS* gene pairs

In addition, we analyzed the duplication events of the *FtGRAS* genes because gene duplication plays an important role in the occurrence of new functions and the amplification of the gene family (Fig. 3; Fig. 4). Chromosomal regions within a 200 kb range of two or more genes were defined as tandem duplication events [39]. Twelve *FtGRAS* genes were clustered into eight tandem duplication event regions in Tartary buckwheat chromosomes 1, 2, and 3, indicating that they were hot spots for *FtGRAS* gene distributions (Fig. 3). Ft1 had three clusters (*FtGRAS1*/*FtGRAS2, FtGRAS4*/*FtGRAS5, FtGRAS5*/*FtGRAS6*), Ft2 also had three clusters (*FtGRAS13*/*FtGRAS14, FtGRAS14*/*FtGRAS16, FtGRAS15*/*FtGRAS16*), and Ft3 had two clusters (*FtGRAS26*/*FtGRAS27, FtGRAS27*/*FtGRAS28*). At the same time, five pairs of segmental duplication events were detected between 5 chromosomes: Ft1 (*FtGRAS112*)/Ft2 (*FtGRAS21*), Ft1 (*FtGRAS11*)/Ft3 (*FtGRAS26*), Ft1 (*FtGRAS3*)/Ft5 (*FtGRAS33*), Ft3 (*FtGRAS25*)/Ft7 (*FtGRAS43*) and Ft7 (*FtGRAS43*)/Ft7 (*FtGRAS46*) (Fig. 4). There were no segmental duplication gene pairs on Ft4, 6, 8. In conclusion, the *FtGRAS* gene tandem duplication and segmental duplication events occurred mainly in HAM and LISCL. Simultaneously, we carried out a synteny analysis of the Tartary buckwheat GRAS genes (Fig. 4). Most of the genes in Tartary buckwheat were kept in collinear blocks, suggesting that the *GRAS* gene family of Tartary buckwheat had a high degree of retention on the corresponding chromosomes during evolution [40]. Concisely, these results suggested that certain *FtGRAS* genes may have been produced by gene duplication and that tandem duplication events may have been the main driving force of *FtGRAS* evolution.

### Evolutionary analysis of *FtGRAS* genes and *GRAS* genes of several different species

**Fig. 5:**
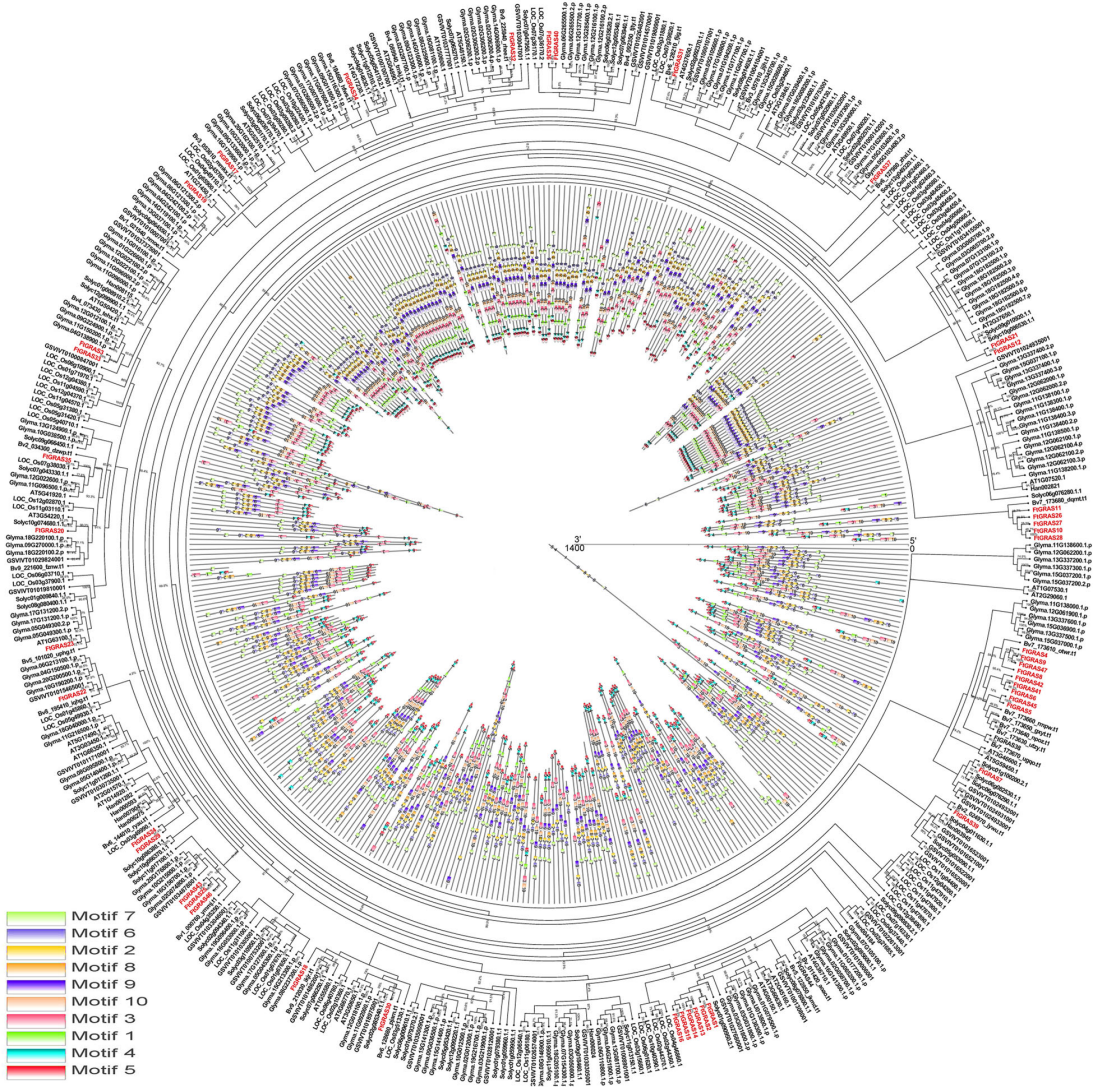
Phylogenetic relationships and motif compositions of GRAS proteins from eight different plant species. The GRAS genes from Tartary buckwheat and other plant species are marked in red and black, respectively. The percentages beside all branches are bootstrap support values generated from 1000 replicates. The motifs, numbered 1–10, are displayed in different colored boxes. The sequence information for each motif is provided in Additional file 4: Table S2

Based on the existing Tartary buckwheat *GRAS* genes, the diversity of the *GRAS* gene family during evolution Was further studied. A phylogenetic tree was constructed using the GRAS protein sequences of seven dicotyledonous plants (*Arabidopsis thaliana*, beet, soybean, grape, tomato, sunflower and Tartary buckwheat) and one monocotyledonous plant (rice). Concurrently, the motifs of the 8 plant GRAS proteins were determined (Fig. 5; Additional file 4: Table S2).

The number of *GRAS* gene and genome size of seven species were soybean (139, 1.025 Gb) [41], rice (60, 389.77 Mb) [42], tomato (53, 900 Mb) [43], Tartary buckwheat (47, 489.3 Mb) [38], grape (43, 427.2 Mb) [44], *Arabidopsis thaliana*, (32, 125 Mb) [45], beet (28, 394.6 Mb) [46], and sunflower (9, 3.6 Gb) [47], respectively. Among the seven species, *Arabidopsis thaliana* has the smallest genome, but the number of GRAS genes was not the least; sunflower had the largest genome, but the number of GRAS genes was not the largest. Therefore, there is no positive correlation between genome size and the number of GRAS genes of these species. We also used MEME web servers to search for conserved motifs that were shared among the *GRAS* proteins, and ten different conserved motifs were found (Motif 1–10) (Fig. 5; Additional file 4: Table S2). Arrangement of the motifs according to the sequence of domains showed that motif 7 belonged to the LHRI domain, motifs 6, 2, and 8 to the VHIID domain, motif 9 to the LHRII domain, motifs 3 and 1 to the RFYRE domain, and motif 5 to the SAW domain. Motif 10 was distributed between motif 9 and motif 3, and motif 4 was distributed between motif 1 and motif 5. Almost all GRAS proteins contained motif 7. *GRAS* members in the same clade, especially the most closely related members, usually shared common motifs, indicating potential functional similarities between GRAS proteins.

**Fig. 6:**
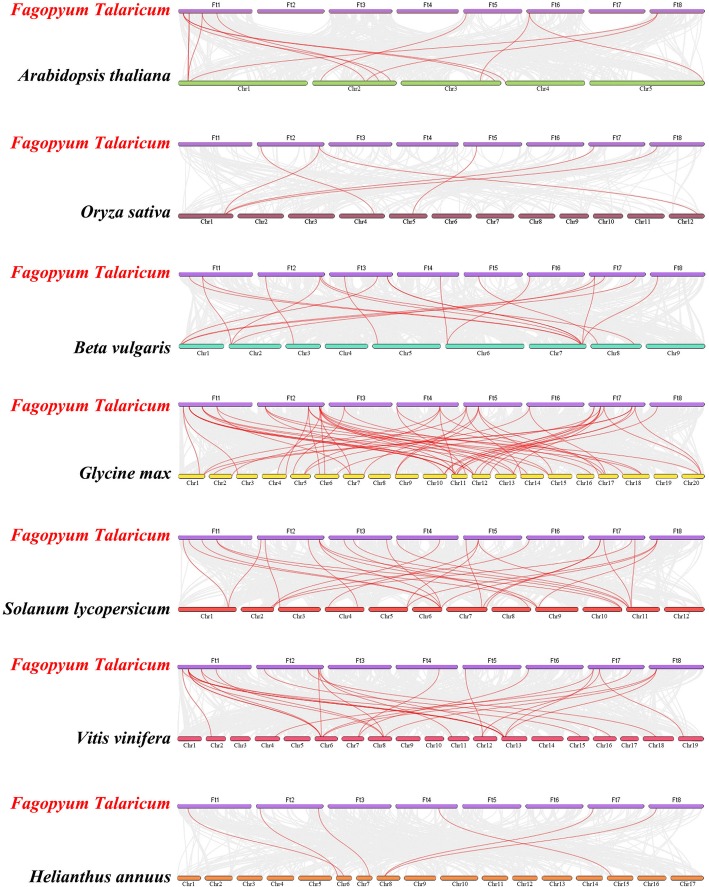
Synteny analysis of the *GRAS* genes between Tartary buckwheat and seven representative plant species. Gray lines in the background indicate the collinear blocks within Tartary buckwheat and other plant genomes, while red lines highlight the syntenic *FtGRAS* gene pairs

To further deduce the phylogenetic mechanism of the Tartary buckwheat *GRAS* gene family, we constructed seven representative comparative systematic maps with Tartary buckwheat, including six dicotyledonous plants (*Arabidopsis thaliana*, soybean, grape, tomato, beet and sunflower) and one monocotyledonous plant (rice) (Fig. 6; Additional file 5: Table S3). A total of 27 *FtGRAS* genes showed syntenic relationships with those in soybean, followed by tomato (21), grape (18), beet (16), *Arabidopsis thaliana* (8), sunflower (6) and rice (5). The number of homologous pairs of the other 7 species (soybean, tomato, grape, beet, *Arabidopsis thaliana*, sunflower and rice) were 57, 29, 28, 19, 13, 6 and 6, respectively. The *FtGRAS* gene had the most syntenic gene pairs with soybean, *FtGRAS19, FtGRAS31, FtGRAS34,* and *FtGRAS40* had four syntenic gene pairs with soybean, and *FtGRAS21* had six syntenic gene pairs with soybean. In addition, *FtGRAS21* had syntenic genes with *GRAS* genes in another five plants (tomato, grape, beet, sunflower and rice), suggesting an important role of *FtGRAS21* in gene evolution.

### Expression patterns of the *FtGRAS* genes in different plant tissues

**Fig. 7:**
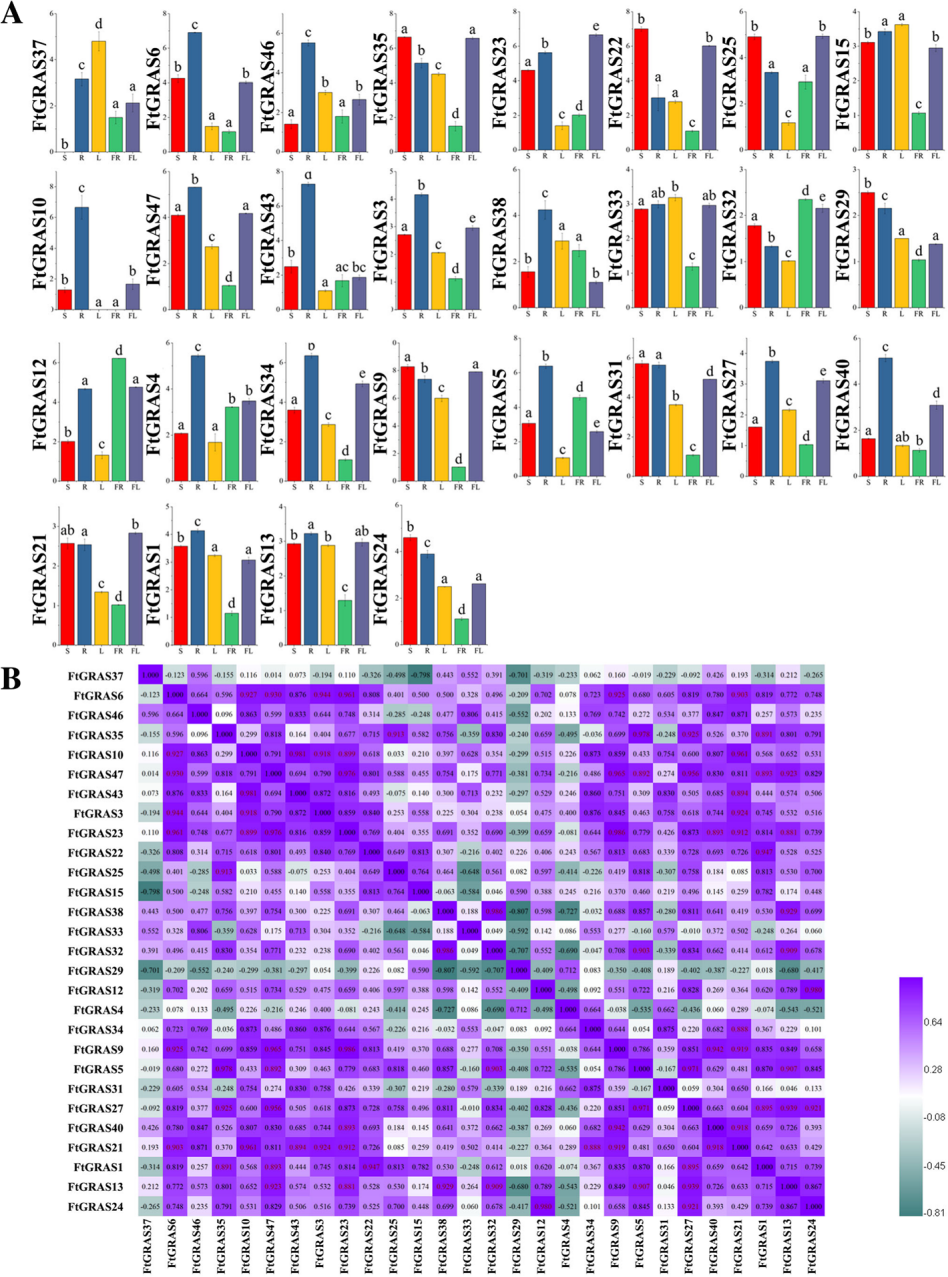
Tissue-specific gene expression of 28 Tartary buckwheat *GRAS* genes. (A) The expression patterns of 28 Tartary buckwheat *GRAS* genes in the flower, leaf, root, stem and fruit tissues were examined by a qPCR assay. Error bars were obtained from fifteen measurements. Lowercase letter(s) above the bars indicate significant differences (α = 0.05, LSD) among the treatments. (B) The correlation between the gene expression patterns of *FtGRAS*. Purple: positively correlated; green: negatively correlated

An evolutionary analysis of the *FtGRAS* gene of several different species was carried out, and 28 genes that may have potential research value were selected (Fig. 7; Additional file 5: Table S4;). To investigate the physiological role of these *FtGRAS* genes, real-time PCR was used to analyze the transcription products of the 28 *FtGRAS* genes in the root, stem, leaf and flower (Fig. 7a). Most of the genes were highly expressed in root, 4 genes (*FtGRAS9, FtGRAS22, FtGRAS25, FtGRAS35*) were highly expressed in both stem and flower, 2 genes (*FtGRAS12* and *FtGRAS32*) were highly expressed in fruit, and 2 genes (*FtGRAS21 and FtGRAS23*) were highly expressed in flower. We also found that *FtGRAS10* was not expressed in stem and *FtGRAS37* was not expressed in leaf and fruit. The results showed diverse transcriptional abundance of *FtGRAS* genes in different tissues and organs, indicating that the *FtGRAS* genes had multiple functions in the growth and development of Tartary buckwheat.

Concomitantly, we analyzed the correlations among the *FtGRAS* gene expression patterns (Fig. 7b). A large proportion of *FtGRAS* gene expression was positively correlated, and some *FtGRAS* genes, such as *FtGRAS24*/*FtGRAS27* (0.921), *FtGRAS12/FtGRAS24* (0.980), and *FtGRAS1/FtGRAS22* (0.947), were significantly correlated.

### Differential expression of *FtGRAS* genes during fruit development of Tartary buckwheat

**Fig. 8:**
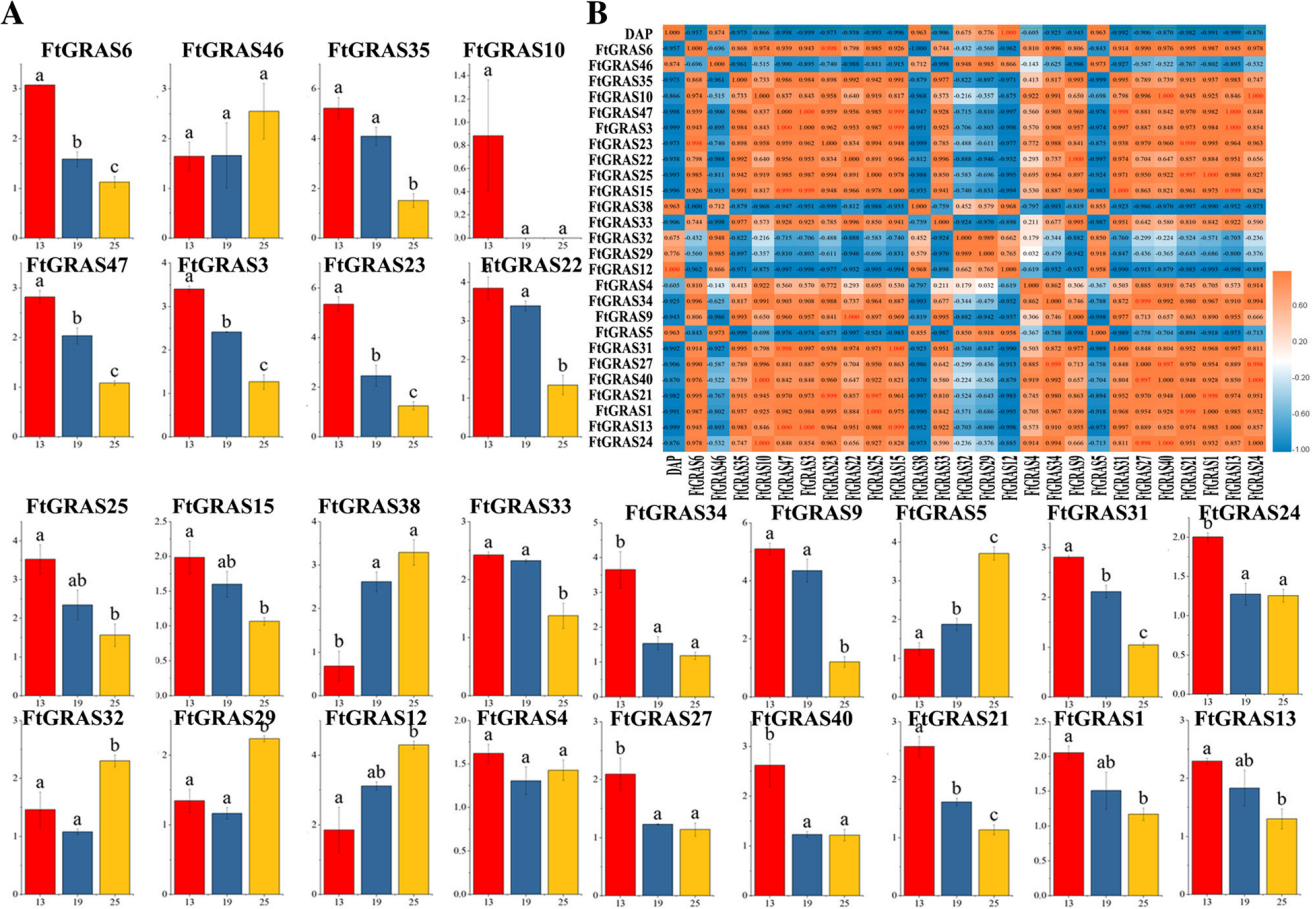
Gene expression of 26 Tartary buckwheat *GRAS* genes during fruit development. (A) The expression patterns of Tartary buckwheat *GRAS* genes in the fruit development stage were examined using a qPCR assay. Error bars were obtained from fifteen measurements. Small letter(s) above the bars indicate significant differences (α = 0.05, LSD) among the treatments. (B) The correlation between the gene expression of *FtGRAS* during fruit development. Red: positively correlated; blue: negatively correlated

The main edible part of Tartary buckwheat is the fruit, which is known for its high content of rutin. Rutin can effectively prevent liver damage and cardiovascular and cerebrovascular diseases [48]. A few reports have examined the gene regulatory networks that regulate the physiological changes during the development of Tartary buckwheat fruit that are supported by the genome of Tartary buckwheat. Therefore, it is important to study the expression patterns of *FtGRAS* genes during the development of Tartary buckwheat fruit. By exploring the expression patterns of the *FtGRAS* gene in different plant tissues, we further selected 26 genes that might be related to fruit development (Fig. 8a). According to previous reports, the green fruit stage (8–14 DAP), discoloration stage (14–22 DAP), and initial maturity stage (22–26 DAP) represent the early, middle and late stages of buckwheat fruit development, respectively [49]. We used real-time PCR to detect the expression of the 26 *FtGRAS* genes at 13, 19 and 25 days after pollination (DAP) (Fig. 8a). The results showed that most of the genes were highly expressed at 13DAP, and 4 genes (*FtGRAS5, FtGRAS12, FtGRAS29, FtGRAS32*) were highly expressed at 25DAP. Two genes (*FtGRAS4, FtGRAS46*) maintained a relatively stable expression level during fruit development.

*FtGRAS* gene expression was negatively correlated with fruit development, except for *FtGRAS5, FtGRAS12, FtGRAS29, FtGRAS32, FtGRAS38,* and *FtGRAS46*. By analyzing the correlations among the *FtGRAS* gene expression patterns (Fig. 8b), we found that most the *FtGRAS* gene expression was positively correlated, and some *FtGRAS* genes, such as *FtGRAS24/FtGRAS27* (0.997), *FtGRAS24/FtGRAS40* (1.000), and *FtGRAS9/FtGRAS22* (1.000) were significantly correlated.

### Expression of DELLA subfamily genes after paclobutrazol treatment

DELLA protein, as the main negative regulator of GA signal transduction, may play an important role in the development of Tartary buckwheat fruit [22, 50]. To further study the relationship between DELLA subfamily genes (*FtGRAS22, FtGRAS24, FtGRAS29*) and fruit development of Tartary buckwheat, we first measured the changes in endogenous GA. Then, we applied paclobutrazol, a triazole plant growth regulator and an inhibitor of endogenous GA synthesis, to affect fruit development [51, 52].

**Fig. 9:**
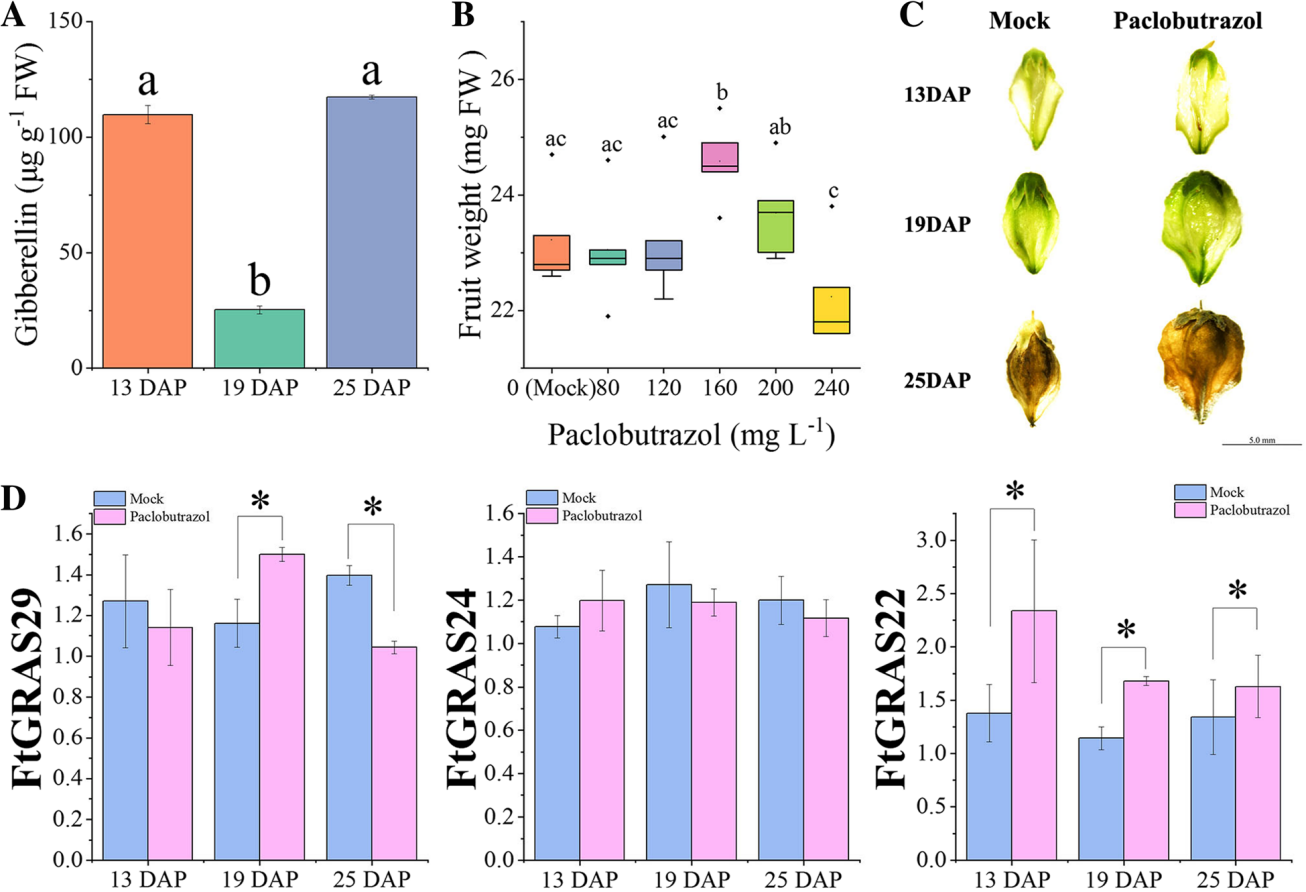
Fruit development of Tartary buckwheat under exogenous paclobutrazol treatment. (A) GA content during fruit development. (B) Final weight of fruits treated with different concentrations of exogenous paclobutrazol. x-axis: weight of mature fruit, y-axis: concentration of paclobutrazol treatment. (C) Images of fruits treated with exogenous paclobutrazol during fruit development. (D) Differences in the expression of DELLA subfamily genes under exogenous paclobutrazol treatment during fruit development. Mock: the same amount of water treatment, paclobutrazol: 160 mg·L^− 1^ paclobutrazol treatment. Error bars were obtained from fifteen measurements. Small letter(s) above the bars indicate significant differences (α = 0.05, LSD) among the treatments. * indicate significant correlations at the 0.05 and 0.01 levels, respectively

We found that the endogenous GA content decreased from 13 to 19 DAP and increased at 19–25 DAP (Fig. 9a). Different concentrations of paclobutrazol (80, 120, 160, 120, and 240 mg L^− 1^) were sprayed on Tartary buckwheat at the bud stage (Fig. 9b). The results showed that the fresh weight of mature fruit increased significantly to 24.58 mg after 160 mg L^− 1^ paclobutrazol treatment, which was 106% of the blank group (23.22 mg). When the concentration of paclobutrazol was higher or less than 200 mg L^− 1^, there was no significant effect on fruit weight gain, and concentration that too high would reduce fruit weight (Fig. 9b). After spraying 160 mg L^− 1^ paclobutrazol, the fruit size increased during the whole fruit development stage (Fig. 9c). We then further explored the effect of exogenous application of 160 mg L^− 1^ paclobutrazol on the expression of the DELLA subfamily genes (*FtGRAS22, FtGRAS24, FtGRAS29*). Paclobutrazol treatment (5 mL) was used as the experimental group and the same amount of water treatment as the control group. The changes in expression of DELLA genes (*FtGRAS22, FtGRAS24, FtGRAS29*) under different treatments were compared (Fig. 9d). The expression levels of the three genes in the fruit development stage changed greatly after treatment with exogenous paclobutrazol. Compared with the control group, *FtGRAS29* expression decreased at 13DAP, increased significantly at 19DAP, and decreased significantly at 25DAP. *FtGRAS24* expression increased at 13 DAP but decreased at 19 DAP and 25 DAP. It is worth noting that after treatment with exogenous paclobutrazol, the expression of *FtGRAS22* increased significantly during the whole fruit development stage. In summary, among the three genes, the responses of *FtGRAS22* and *FtGRAS29* to external paclobutrazol were more obvious, especially *FtGRAS22*.

## Discussion

GRAS transcription factors play vital roles in regulating plant growth and development [12]. With the development of biotechnology and bioinformatics, the *GRAS* gene family has been identified and analyzed in many plants, such as *Arabidopsis thaliana*, rice, tomato, and pepper. The whole genome sequence of Tartary buckwheat has been identified, but there are few reports on *GRAS* in Tartary buckwheat. In this study, we systematically analyzed the *GRAS* gene family in Tartary buckwheat.

### *FtGRAS* gene structure and evolutionary analysis

A total of 47 *FtGRAS* genes were obtained from the Tartary buckwheat genome. The number of *FtGRAS* genes in Tartary buckwheat is higher than that in *Arabidopsis thaliana* (32) [10], close to that in Chinese cabbage (48) [37], castor beans (48) [53] and pepper (50) [2], and less than that in tomato (53) [34], rice (60), [10], and poplar (106), [11]. Some studies have shown that the origin of the plant *GRAS* gene family derived from the prokaryotic genome through horizontal gene transfer, followed by duplication events [54]. Therefore, the changes in the number of *GRAS* genes may be associated with gene duplication events. Based on previous research, 2/34, 15/53, 12/50 17/60, and 40/106 were identified as tandem duplicated genes in *Arabidopsis thaliana* [10], tomato [34], pepper [2], rice [10], and poplar [11], respectively. The gene duplication events may increase the number of *GRAS* genes in these five plants. In this study, eight pairs of tandem duplication *FtGRAS* genes and five pairs of segmental duplication *FtGRAS* genes were detected (Fig. 3; Fig. 4). Our results further verify the effect of duplication on the expansion of the *GRAS* gene family. The contribution of tandem duplication to the expansion of Tartary buckwheat *GRAS* was greater than that of segmental duplication. Interestingly, we found some differences in the expression patterns of tandem duplicated genes (*FtGRAS5 and FtGRAS6*). The expression of *FtGRAS6* in fruit was obviously lower than that of *FtGRAS5*. We further analyzed their expression during fruit development and found that the expression of *FtGRAS6* decreased gradually with the development of fruit, but the expression pattern of *FtGRAS5* was completely the opposite (Fig. 7a; Fig. 8a). Comparison of motifs found that they contained the same motifs (motif 6, 7, 1, 4, 9, 3, 2, 5). By comparing their 3D structures, we observed a different random coil in the center of FtGRAS5 and FtGRAS6 protein (Additional file 1: Figure S 1). Further comparison of their secondary structure showed mainly a difference in the random coil (Additional file 2: Figure S2). Therefore, the difference in the secondary structure or tertiary structure may lead to the functional difference of the protein. The duplicated genes may undergo different processes of selection (nonfunctionalization, neofunctionalization, or subfunctionalization), which may result in the diversity expression patterns or protein structures [55]. Therefore, we speculate that the two genes (*FtGRAS5 and FtGRAS6*) may have undergone different evolutionary processes, resulting in differences in protein structures and expression patterns during fruit development.

By analyzing the intron-exon structure of *FtGRAS* genes, we found that most of the *FtGRAS* genes (41, ~ 87%) had no intron structure, which was similar to the results of tomato (77%) [34], *Prunus mume* (83%) [56], Chinese cabbage (83%) [37], pepper (84%) [2], and grapevine (88.46%) [57]. The increase in genomic data and the establishment of more reliable models showed that the ancestors of each eukaryotic supergroup had intron-rich genes. The subsequent evolution of most eukaryotes involved the loss of introns [58]. The high proportion of intron-free genes in these plants (tomato, *Prunus mume*, Chinese cabbage, pepper, grapevine and Tartary buckwheat) suggests that they may have experienced intron loss events during evolution.

The domains and motifs of transcription factors play an important role in protein interaction and DNA binding [59]. GRAS contains five conserved domains at the C-terminus, all of which have important functions [4]. Ten different motifs were identified in Tartary buckwheat (Fig. 2c),and most of the *FtGRA*S genes contained motifs 6, 7, 1, 9, 3, 2, and 5. However, only LISCI, SHR, and PAT1 contained motif 4 (belonging to LHRII), which may mediate protein-protein interactions [4], suggesting that these subfamilies may have unique functions. Outside the domains of GRAS, we also found some conserved motifs, such as motif 10 (Fig. 2c, Additional file 4: Table S2), motifs 4 and 10 (Fig. 5, Additional file 4: Table S2). Motif 10 was only found in the HAM subfamily (Fig. 2c, Additional file 4: Table S2), which might confer a distinctive function to this subfamily.

### Predicting the potential functions of *FtGRAS* genes

Intrinsically disordered proteins (IDPs) are biologically active proteins that do not form a fixed 3D structure under physiological conditions [60, 61]. Depending on the particularities of the environment, an IDP can be folded into different structures to identify and bind diverse partners at different binding interfaces [62, 63]. With the use of computation and bioinformatics, it has been proven that GRAS protein is intrinsically disordered [60, 64]. A typical IDP of the GRAS protein is its highly variable N-terminus, which has short interacting fragments and molecular recognition features that are responsible for recognizing and binding specific partners to GRAS proteins [8]. IDP may lead to the functional differences in FtGRAS proteins.

By analyzing the expression pattern of *FtGRAS* gene in different plant tissues, we found a diverse transcript abundance of *FtGRAS* genes in different tissues and organs (Fig. 7a), implying that they might differ in function. Additionally homologous genes may have similar functions. *FtGRAS23* showed a higher expression level in the flower, which is consistent with the expression pattern of the homologous gene *ATSCL28* (*AT1G63100*), and *SCL28* may play a sperm-specific role in *Arabidopsis thaliana* [65, 66]*.* The transcription level of *FtGRAS35* and the homologous gene *ATSCL23 (AT5G41920)* were both high in flower; ATSCL23 and SHR form negative a feedback loop, and the SHR-SCR-SCL23 module plays a key role in the formation of endodermis in *Arabidopsis thaliana* [66, 67]. *FtGRAS32* is one of the gene that are homologous to the *Arabidopsis thaliana* gene *PAT1* (*AT5G48150.1*). *FtGRAS32* and *PAT1* are highly expressed in fruit, and PAT1 is involved in PhyA signal transduction [9, 66]. Simultaneously, we also found that most *FtGRAS* members expressed higher levels in early fruit development, which gradually decreased with fruit development. This situation is similar to *GRAS* in castor beans, indicating that these genes may be involved in early fruit development [53].

### DELLA subfamily and fruit development of Tartary buckwheat

The DELLA subfamily is widely and extensively studied in the *GRAS* gene family, which are key regulators of the GA signal transduction pathway. DELLA protein inhibits plant growth and development, while GA promotes plant growth and development by degrading DELLA protein [68]. Degradation of DELLA is initiated by the formation of the GA-GID1-DELLA complex, which is then identified by the specific ubiquitin E3 ligase complex (SCFSLY1/GID2), which marks the DELLA protein for degradation by the 26S proteasome [68]. DELLA also plays an important role in the development of fruit [22–24]. To initially explore the relationship between GA and DELLA in Tartary buckwheat, we determined the content of endogenous GA during the development of buckwheat fruit (Fig. 8a), which decreased gradually from 13 to 19 DAP, reached the lowest value at 19 DAP, and then increased in the late stage of fruit development (19–25 DAP). Compared with the expression level of DELLA subfamily members (*FtGRAS22*, *FtGRAS24*, *FtGRAS29*), the expression level of *FtGRAS22* in late fruit development (19~25 DAP) was opposite to that of GA. GA plays an important role in the late embryonic development of *Arabidopsis thaliana*, and the change in GA content in late embryonic development is opposite to the DELLA protein content [22]. Therefore, we speculate that *FtGRAS22* may play a role in late fruit development. Interestingly, rice DELLA protein SLR1 (LOC-OS03g49990.1) can regulate cellulose synthesis by interacting with NAC transcription factors [25]. Cellulose is an important component of the plant cell wall, which can affect the cracking of Tartary buckwheat fruit [69, 70]. We found that FtGRAS24 and FtGRAS29 were highly homologous to SLR1(Fig. 5), so we speculated that FtGRAS24 and FtGRAS29 might have similar functions to SLR1.

To further explore the relationship between DELLA genes and Tartary buckwheat fruit development, we sprayed paclobutrazol on the plants, a triazole plant growth regulator; the main biochemical function of paclobutrazol is to inhibit GA biosynthesis. The target enzyme of paclobutrazol is ent-kaurene oxidase (KO), which specifically inhibits the oxidation of ent-kaurene to ent-kaurene acid, thus hindering the synthesis of GA [71]. Then main morphological effects of paclobutrazol on plant growth include shortening the internode length, reducing the leaf area, increasing the leaf thickness, and increasing the flower bud number and fruit setting rate [72]. In this experiment, we observed a significant increase in the weight of Tartary buckwheat fruit by exogenous application of 160 mg L^− 1^ paclobutrazol (Fig. 8b, c). We further measured the expression changes of the DELLA subfamily (*FtGRAS22, FtGRAS24, FtGRAS29*) upon treatment with paclobutrazol, an inhibitor of GA synthesis (Fig. 8d). Compared with the control group, the change in *FtGRAS24* was not obvious, which suggested *FtGRAS24* might not be sensitive to GA. *FtGRAS29* increased significantly at 19 DAP, but it decreased significantly at 25DAP. The expression level of *FtGRAS22* increased significantly during the whole fruit development period after paclobutrazol treatment. In conclusion, the sensitivity of *FtGRA22* to GA was stronger than *FtGRS24* and *FtGRAS29*. Thus, we speculate that *FtGRAS22* may have an important impact on the development of Tartary buckwheat fruit and can be used as a candidate gene for Tartary buckwheat breeding.

Based on a preliminary analysis of DELLA subfamily members (*FtGRAS22, FtGRAS24, FtGRAS29*) in Tartary buckwheat, we found that the expression levels of the three genes and the sensitivity to GA during fruit development varied widely, suggesting that the three genes might be functionally different. DELLA protein can also be post-translationally modified by phosphorylation and *O*-Glc-Nac modification [73–77]. Post-translational modifications may lead to differences in function. Therefore, members of the DELLA subfamily in the same plant may also differ in function, and the mechanisms of DELLA protein and GA signal transduction in the process of Tartary buckwheat fruit development deserve further study.

## Conclusions

In this study, 47 *GRAS* genes of Tartary buckwheat were identified and divided into 10 subfamilies: LISCL, HAM, DELLA, SCR, PAT1, SCL4/7, LAS, SHR, SCL3, and DLT. By analyzing the gene structure of *FtGRAS*, we found that most genes did not contain introns. Certain *FtGRAS* genes might have been produced by gene duplication; tandem duplication contributed more to the expansion of the *GRAS* gene family in Tartary buckwheat than did segmental duplication. The expression patterns of *FtGRAS* genes in different tissues (root, stem, leaf, flower, fruit) and fruit development stages (13, 19, 25 DAP) were further studied, indicating that they might have different functions in the growth and development of Tartary buckwheat. Furthermore, the increase in fruit weight was induced by exogenous paclobutrazol, and the transcription level of the DELLA subfamily member *FtGRAS22* was significantly upregulated. Therefore, *FtGRAS22* might be a potential target for molecular breeding or genetic editing. In conclusion, these findings provide a theoretical basis for studying the potential function of Tartary buckwheat *GRAS* genes.

## Methods

### Plant growth

The Tartary buckwheat variety used in this experiment was XIQIAO, which is a high rutin variety of Tartary buckwheat obtained by physical and chemical mutagenesis [78]. The Tartary buckwheat (XIQIAO) used in this study was provided by Professor Wang Anhu of Xichang University. From 2013 to 2018, XIQIAO was introduced into the experimental field of the College of Life Science, Sichuan Agricultural University, Ya’an, Sichuan, China, and the ecological environment and cultivation conditions were the same during those years. The materials were collected in 2017. The samples, including stem, root, leaf, flower and fruit at 13, 19, and 25 DAP were collected separately from 5 plants with good growth and similar growth conditions, and quickly placed in liquid nitrogen and stored at − 80 °C for further use [27].

### Identification of *GRAS* genes in Tartary buckwheat

Genes identification referred to the method of Liu et al. [27]. The genome of Tartary buckwheat was obtained from the Tartary Buckwheat Genome Project (TBGP; http://www.mbkbase.org/Pinku1/). First, all known AtGRAS proteins were used to query the initial protein on the TBGP website by BLASTp. Second, we downloaded the hidden Markov model (HMM) file corresponding to the GRAS domain (PF03514) from the Pfam protein family database (http://pfam.sanger.ac.uk/). The GRAS protein sequences of Tartary buckwheat were aligned using the HMM model in HMMER3.0. The existence of the GRAS core sequences was verified by the PFAM and SMART programs. Forty-seven *GRAS* genes were identified in the Tartary buckwheat genome. Finally, 47 FtGRAS proteins were used as initial queries in the NCBI protein database (https://blast.ncbi.nlm.nih.gov/Blast.cgi? PROGRAM = blastp&PAGE_TYPE = BlastSearch&LINK_LOC = blasthome) by BLASTp, further verifying that 47 proteins derived from Tartary buckwheat belonged to the GRAS gene family. The tools from the ExPASy website (https://web.expasy.org/protparam/) were used to obtain the sequence length, molecular weight, and isoelectric point of the identified GRAS protein. The subcellular localization of GRAS protein was predicted using CELLO (http://cello.life.nctu.edu.tw/).

### Phylogenetic analysis and classification of *FtGRAS* genes

The phylogenetic trees were derived using the Maximum Likelihood (ML) method. The AtGRAS and FtGRAS amino acid sequences were aligned using MUSCLE [79]. We used the JTT + G + F model to identify the best-scoring tree in MEGA 7 [80]. The ML phylogenetic tree was constructed with 1000 bootstraps replicates. According to the classification of *AtGRAS*, all the identified *FtGRAS* genes were divided into different groups. The GRAS protein sequences (*Arabidopsis thaliana*, beet, soybean, grape, tomato, rice, sunflower) were downloaded from the UniProt database (https://www.uniprot.org), and phylogenetic trees were constructed with the above-described ML method.

### Gene structure and conserved motif analysis

We used the gene structure display server (GSDS: http://gsds.cbi.pku.edu.cn) online program to analyze the exon-intron structure of the *FtGRAS* genes based on the CDS and the corresponding full-length sequence. The conserved motifs were studied in the encoded GRAS proteins to investigate the structural differences between the *FtGRAS* genes. We used the GRAS domain sequence of the FtGRAS proteins and default parameters of ClustalW to compare protein sequences. A MEME online program (http:/meme.nbcr.net/meme/intro.html) was used to analyze the protein sequences under the following parameters: the optimum motif width was 15 ~ 50, and the maximum number of motifs was 10.

### Chromosomal distribution and gene duplication of *FtGRAS* genes

The physical location information was obtained from the Tartary buckwheat genomic database by Circos, and all *FtGRAS* genes were mapped to the chromosomes of Tartary buckwheat. Multiple collinear scanning toolkits (MCScanX) with default parameters were used to analyze gene duplication events. The syntenic relationship between *FtGRAS* genes and *GRAS* genes from selected plants was determined using Dual Synteny Plotter software.

### Structural prediction using protein modeling

Putative protein sequences of 47 FtGRAS were used as query sequences. The secondary and tertiary structures of FtGRAS Protein were built using SOPMA (https://npsa-prabi.ibcp.fr/cgi-bin/npsa_automat.pl?page=npsa_sopma.html) and SWISS-MODEL (https://www.swissmodel.expasy.org/interactive), respectively.

### Endogenous GA analysis and Paclobutrazol treatments

The determination method of endogenous GA in Tartary buckwheat was as follows: first, 0.5 ± 0.01 g of fresh sample was collected and quickly ground in liquid nitrogen. The ground powder was homogenized in 80% methanol (10 mL) and stirred at 4 °C overnight. The supernatant was collected after centrifugation at 13900 x *g* for 10 min at 4 °C. The precipitate was washed once more by adding 80% methanol (5 mL), and the supernatant was collected after centrifugation. The mixed supernatant was evaporated at 36 °C until no methanol remained. Second, 5 mL ultrapure water was used to wash the rotary evaporator bottle, and the rinsing solution was combined with the residual liquid. The solution was decolorized with diethyl ether three times, and then the water phase was collected and alkalized to pH 8.0. Next, the alkalized extract was mixed with 50 mg of polyvinylpyrrolidone and then shaken for 30 min. The supernatant was collected by centrifugation at 13900 x *g* for 10 min at 4 °C, followed by acidification to pH 3.0 with citric acid. The ethyl acetate phase was collected by separating the solution three times with 5 mL of ethyl acetate. The combined ethyl acetate phases were evaporated to near dryness at 36 °C. Finally, the residue was dissolved in 1 mL of methanol. The final samples were analyzed by HPLC [27, 81, 82].

Paclobutrazol is a triazole plant growth regulator that can inhibit the concentration of GA in plants and is widely used in horticultural crops to shorten the internode length, reduce the leaf area, increase leaf thickness, and increase flower bud number and fruit set rate [83]. XIQIAO with similar growth statuses were selected and sprayed with 5 mL of different concentrations of paclobutrazol (80, 120, 160, 120 and 240 mg·L^− 1^) during the germination period. The same amount of water was sprayed as a blank control. Fruit samples were collected at 13, 19 and 25 DAP, respectively, frozen in liquid nitrogen and stored at − 80 °C for further use.

### Expression analysis of the *FtGRAS* genes by real-time PCR

Total RNA was extracted using the RNAout Kit (TIANGEN, China) and treated with RNase free DNase I to remove trace amounts of DNA. According to the manufacturer’s instructions, the cDNA was pretreated with the PrimeScript™ 1st Strand cDNA Synthesis Kit (Japanese Takara). We obtained the corresponding sequences of these genes from the Tartary buckwheat (Pinku1) genome sequence database (http://www.mbkbase.org/Pinku1/) and then used Primer3 software (http://frodo.wi.mit.edu/) to design the RT-qPCR primers (Additional file 6: Table S4). Using the FtH3 gene as an internal control [84], standard RT-qPCR with SYBR Premix Ex Taq II (TaKaRa) was repeated at least three times on a CFX96 Real-Time System (BioRad). The data were analyzed by the 2^−(∆∆Ct)^ method, and the relative mRNA expression data were obtained [85].

### Statistical analysis

The Origin Pro 2019b (OriginLab Corporation, Northampton, Massachusetts, USA) statistics program was used to analyze all the data by analysis of variance, and the means were compared by the least significant difference test (LSD) levels of significance.

## Additional files

Additional file 1: **Figure S1.** Prediction of the 3D structures of 47 FtGRAS protein. (PNG 15359 kb)

Additional file 2: **Figure S2.** Secondary structures prediction of FtGRAS5 and FtGRAS6. (PNG 2066 kb)

Additional file 3: **Table S1.** List of the 47 *FtGRAS* genes identified in this study. (XLS 153 kb)

Additional file 4: **Table S2.** Analysis and distribution of conserved motifs in Tartary buckwheat GRAS proteins. (XLS 36 kb)

Additional file 5: **Table S3.** One-to-one orthologous relationships between Tartary buckwheat and other plants. (XLS 82 kb)

Additional file 6: **Table S4.** The primer sequences used for RT-qPCR. (XLS 39 kb)

## Data Availability

The genome sequences of Tartary buckwheat used to identify the *GRAS* genes in this study are located in the Tartary Buckwheat Genome Project (TBGP; http://www.mbkbase.org/Pinku1/). The Tartary buckwheat accessions (XIQIAO; accessions number: CHUAN 2008013) used in the experiment were supplied by Professor Wang Anhu of Xichang University. These plant materials are widely used all over the world. and no permits are required for the collection of plant samples. The datasets supporting the conclusions of this article are included with in the article and its Additional files.

## Abbreviations

At: *Arabidopsis thaliana*
BLASTs: Basic local alignment search tools
CDS: Coding sequence length
DAP: Days after pollination
Ft: *Fagopyrum tataricum*
GA: Gibberellin
GAI: Gibberellic acid insensitive
IDPs: Intrinsically disordered proteins
LHRI: Leucine-rich region I
LHRII: Leucine-rich region II
LSD: Least significant difference test
MCScanX: Multiple collinear scanning toolkits
ML: Maximum Likelihood
Mw: Protein molecular weight
phyA: Phytochrome A
pI: Isoelectric point
RGA: Repressor of GA1–3 mutant
SCR: Scarecrow
TFs: Transcription factors
