# Supplementary figures and images for: Genome-wide identification, expression analysis and functional study of the GRAS gene family in Tartary buckwheat (Fagopyrum tataricum)
Source: BMC Plant Biol. 2019 Aug 6;19:342. doi: 10.1186/s12870-019-1951-3 (PMC6683366; doi:10.1186/s12870-019-1951-3)

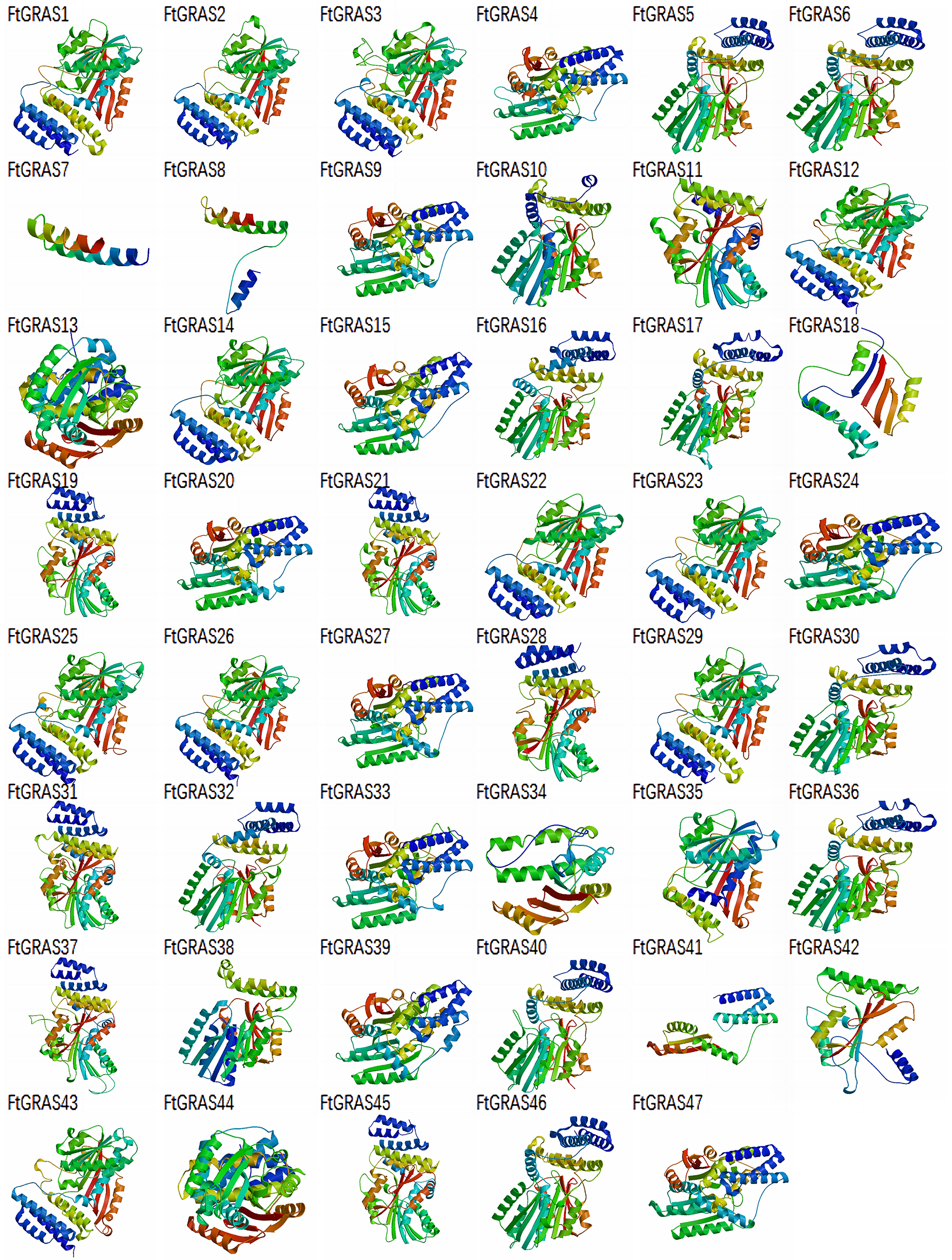

Supplement: Supplementary file 1 — Figure S1. Prediction of the 3D structures of 47 FtGRAS protein. (PNG 15359 kb) [file 12870_2019_1951_MOESM1_ESM.png]

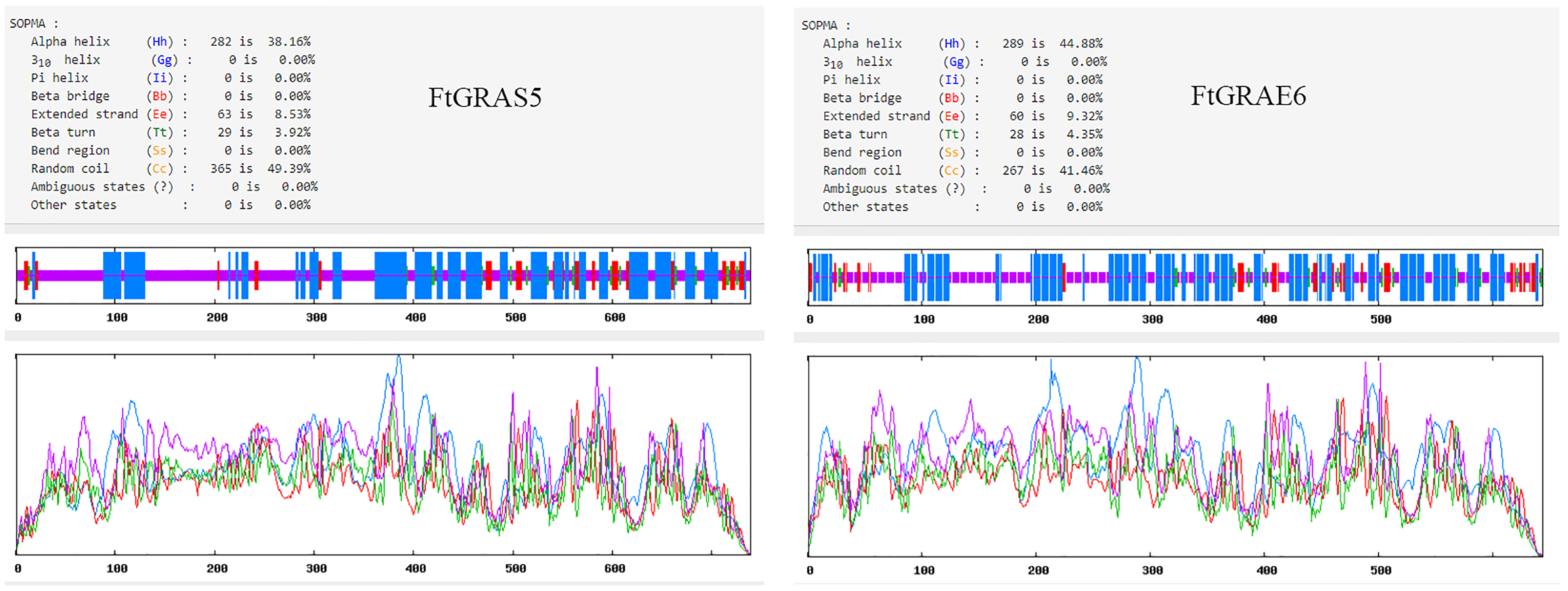

Supplement: Supplementary file 2 — Figure S2. Secondary structures prediction of FtGRAS5 and FtGRAS6. (PNG 2066 kb) [file 12870_2019_1951_MOESM2_ESM.png]
